# Lovastatin induces apoptosis of ovarian cancer cells and synergizes with doxorubicin: potential therapeutic relevance

**DOI:** 10.1186/1471-2407-10-103

**Published:** 2010-03-18

**Authors:** Anna Martirosyan, James W Clendening, Carolyn A Goard, Linda Z Penn

**Affiliations:** 1 Ontario Cancer Institute/Princess Margaret Hospital, Campbell Family Institute for Cancer Research, Toronto, ON, Canada; 2 Department of Medical Biophysics, University of Toronto, Toronto, ON, Canada

## Abstract

**Background:**

Ovarian carcinoma is a rarely curable disease, for which new treatment options are required. As agents that block HMG-CoA reductase and the mevalonate pathway, the statin family of drugs are used in the treatment of hypercholesterolemia and have been shown to trigger apoptosis in a tumor-specific manner. Recent clinical trials show that the addition of statins to traditional chemotherapeutic strategies can increase efficacy of targeting statin-sensitive tumors. Our goal was to assess statin-induced apoptosis of ovarian cancer cells, either alone or in combination with chemotherapeutics, and then determine these mechanisms of action.

**Methods:**

The effect of lovastatin on ovarian cancer cell lines was evaluated alone and in combination with cisplatin and doxorubicin using several assays (MTT, TUNEL, fixed PI, PARP cleavage) and synergy determined by evaluating the combination index. The mechanisms of action were evaluated using functional, molecular, and pharmacologic approaches.

**Results:**

We demonstrate that lovastatin induces apoptosis of ovarian cancer cells in a p53-independent manner and synergizes with doxorubicin, a chemotherapeutic agent used to treat recurrent cases of ovarian cancer. Lovastatin drives ovarian tumor cell death by two mechanisms: first, by blocking HMG-CoA reductase activity, and second, by sensitizing multi-drug resistant cells to doxorubicin by a novel mevalonate-independent mechanism. This inhibition of drug transport, likely through inhibition of P-glycoprotein, potentiates both DNA damage and tumor cell apoptosis.

**Conclusions:**

The results of this research provide pre-clinical data to warrant further evaluation of statins as potential anti-cancer agents to treat ovarian carcinoma. Many statins are inexpensive, off-patent generic drugs that are immediately available for use as anti-cancer agents. We provide evidence that lovastatin triggers apoptosis of ovarian cancer cells as a single agent by a mevalonate-dependent mechanism. Moreover, we also show lovastatin synergizes with doxorubicin, an agent administered for recurrent disease. This synergy occurs by a novel mevalonate-independent mechanism that antagonizes drug resistance, likely by inhibiting P-glycoprotein. These data raise important issues that may impact how statins can best be included in chemotherapy regimens.

## Background

As a malignancy with particularly poor prognosis, novel therapeutic options are urgently required for the treatment of ovarian cancer[1,2] In 2009, approximately 25,000 women will be diagnosed in North America and most will die of their disease, making it the fifth leading cause of cancer mortality in women[3] The majority of ovarian cancer cases present as advanced stage III or IV disease and treatment usually involves surgical cytoreduction, followed by adjuvant platinum/taxane chemotherapy, with about 70-80% response rates. While patients typically undergo a period of remission of 1-2 years, more than half eventually relapse. Some patients with recurrent disease become refractory to platinum treatment. They are generally next treated with regimens of gemcitabine, topotecan, and/or liposomal doxorubicin, but with very limited success[4,5] The reduced rate of response in these patients is typically due to the development of drug resistance[6] Taken together, to directly increase the quality and longevity of life, new and immediate therapeutic approaches are urgently required to combat ovarian cancer.

We and others have shown that the statin family of drugs exhibit antiproliferative activity against cancer cells without causing collateral damage to normal cells[7]. Statins inhibit the rate-limiting enzyme of the mevalonate (MVA) pathway, HMG-CoA reductase (HMGCR), and have been used for decades as safe and effective agents in the control of hypercholesterolemia[7,8] In addition to cholesterol, the MVA pathway gives rise to a number of crucial biochemical end-products, including ubiquinone, dolichol, isopentenyladenine, and isoprenoid precursors. Statins can trigger tumor cells to undergo a classic caspase-dependent, apoptotic response that is reversible by exogenous addition of MVA or the isoprenoid precursors, geranylgeranyl pyrophosphate (GGPP) and farnesyl pyrophosphate (FPP)[7]. Thus, the statin family of drugs are immediately available for use as part of the arsenal of molecular targeted therapeutics to combat cancer.

Like most anti-cancer agents, statins demonstrate robust efficacy on some but not all tumor-types, emphasizing the importance of matching the agent with the sensitive, responsive cancer. Statins have been extensively shown to trigger apoptosis of cell lines derived from haematological malignancies, including acute myelogenous leukemia and multiple myeloma[7,9]. This preclinical data has been recently translated to Phase I/II clinical trials that have shown promising results when statins have been used in combination with standard chemotherapy[10,11] Similarly, median survival was doubled with the addition of statins to 5-fluorouracil in advanced hepatocellular carcinomas[12] As was recently reviewed, solid tumor derived cell lines that have recently been shown to be statin sensitive include breast, colorectal, lung, prostate, and pancreatic, [13]. however, preclinical work focusing on ovarian cancer is required to determine whether statins have the potential to be used to combat this tumor type as well. Very recently, preliminary reports have indicated that ovarian carcinoma is sensitive to statin-induced apoptosis, providing a unique alternative to treating this deadly disease[14,15].

To advance these findings, we demonstrate that lovastatin induces apoptosis of ovarian cancer cells in a p53-independent manner and synergizes with doxorubicin, a chemotherapeutic agent used to treat recurrent ovarian cancer. Lovastatin triggers ovarian tumor cells to undergo apoptosis by two mechanisms: first, by blocking HMGCR activity; and second, by increasing the level of doxorubicin within drug-resistant cells. Together, these data support further pre-clinical and clinical evaluations of statins as a new strategy to combat ovarian cancer and overcome drug resistance.

## Methods

### Cells

Cells were grown as a monolayer in RPMI 1640 medium with 10% fetal bovine serum in a humidified incubator at 37°C in 5% CO_2_. Ectopic expression of the ecotropic receptor was conducted as described[16]. and subsequent gene transfer was achieved by infection with retrovirus produced using the Phoenix ecotropic packaging system as previously described[17] A2780 cells were generated to ectopically express either a p53DD construct or the empty YFP vector control by flow sorting for stably expressing cells[17] Cells were also generated and selected to ectopically express Bcl-2 and its corresponding empty vector control.

### MTT assays

The MTT assays were conducted as previously described[18]. except 3750 cells/well of a 96-well plate were plated and after 24 hours, cells were exposed to lovastatin (5 to 100 μM; activated as previously described[19]) for 48 hours followed by MTT tetrazolium substrate for 2 hours.

### Immunoblotting

Cells were seeded in 100 mm dishes for 24 hours. Treatments of 20 μM lovastatin or vehicle control were completed for 24 or 48 hours before cells were harvested for PARP (Cell Signaling Technology), Rap1 (Santa Cruz Biotechnology), p53 (Santa Cruz Biotechnology), actin (Sigma-Aldrich), and tubulin (Calbiochem) immunoblotting as described[9,17] Lysates from cells exposed to 8 GY of radiation for 8 hours were immunoblotted for p21 (Santa Cruz Biotechnology) and tubulin. Lysates from A2780 pBP, A2780 Bcl-2, A2780ADR GFP and A2780ADR Bcl-2 were immunoblotted for Bcl-2 and tubulin.

### Fixed PI

Cells were seeded in 100 mm dishes for 24 hours and exposed to indicated lovastatin concentrations or vehicle control for indicated times (24, 48, or 72 hours), washed in PBS, fixed in 70% ethanol, stained with 50 μg/mL PI (Sigma-Aldrich) and analyzed by a FACScalibur flow cytometer (BD Bioscience). Ten thousand events were scored and analysis was performed using Cell Quest software (BD Bioscience) to assess the dying, pre-G1 population.

### Synergy experiments

Cells were plated for MTT assays and treated for 24 hours with concentration ranges of lovastatin and either doxorubicin or cisplatin that are centered on each drug's MTT_50 _(A2780ADR - 20, 14, and 42 μM, respectively; A2780 - 10, 4, 50 μM, respectively; A2780CIS - 30, 5, 112 μM, respectively). CEMVBL cells were similarly plated and treated with lovastatin and doxorubicin, also centered on each drug's MTT_50 _(42 and 68 μM, respectively). Drug treatments were performed individually or in fixed ratio combinations (at 4×, 2×, 1×, 1/2×, 1/4×, 1/8× MTT_50 _values) as described previously[20]. Combination index (CI) plots were generated using CalcuSyn software (Ver 2; Biosoft) based on algorithms developed by Chou and Talalay[21] to determine whether the drugs synergize (CI < 1).

### Measurement of P-gp expression

As previously described, [22] 1 × 10^6 ^cells were harvested, washed in 1 mL buffer (1xPBS, 0.5%BSA), resuspended in 1 mL staining buffer (1xPBS, 0.5%BSA, 0.1%NaN3) with or without 20 μL of FITC-labelled anti-human P-gp (BD Pharmigen), and incubated on ice for 40 minutes in the dark. Cells were washed with staining buffer, resuspended in 1 mL buffer, and fluorescence in the FL1 channel was detected by flow cytometry. 10 000 events were captured for analysis with Cell Quest software.

### Measurement of intracellular doxorubicin

Intracellular doxorubicin was measured as described previously[23]. with some alterations. Briefly, for accumulation experiments, 2 × 10^5 ^A2780ADR cells were seeded in 6-well plates for 24 hours and treated. Alternatively, 5 × 10^5 ^CEMVBL cells were seeded and treated in 6-well plates. A2780ADR cells were exposed to 7 μM doxorubicin (half MTT_50_) alone or in combination with 5, 10 or 20 μM lovastatin for 3 hours. An additional sample was treated with doxorubicin, 10 μM lovastatin, and 100 μM MVA as well. CEMVBL cells were exposed to 34 μM doxorubicin (half MTT_50_) alone or in combination with 2.63, 5.25, 10.5 or 21 μM lovastatin for 3 hours. An additional sample was treated with doxorubicin, 21 μM lovastatin, and 100 μM MVA as well. For retention experiments, A2780ADR cells were incubated with a combination of 7 μM doxorubicin and 10 μM lovastatin as above and were further incubated for two hours in doxorubicin-free media with or without 10 μM lovastatin. For both accumulation and retention experiments, after treatment, cells were washed twice in cold phosphate buffered saline (PBS), resuspended in 1 mL of cold PBS, and filtered for single cells on ice. Intracellular doxorubicin fluorescence was measured by detecting the natural fluorescence of doxorubicin with flow cytometry (FL2 channel); 10,000 events were scored.

### Comet assay

A2780ADR cells were seeded in 100 mm plates for 24 hours and exposed to either a control, 10 μM lovastatin, 7 μM doxorubicin, or both 10 μM lovastatin and 7 μM doxorubicin together for 24 hours. Comet assays were performed as described previously[24] Briefly, comets were visualized by fluorescence microscopy (Axioskop microscope, Zeiss) and images were analyzed using Komet 5.5 software (Kinetic Imaging). DNA damage was quantified from the analysis of 75 comets per treatment/experiment using the olive tail moment [Tail moment = (%DNA) × (distance traveled)].

### TUNEL assay

TUNEL and PI dual-staining was carried out as per manufacturer's instructions (APO-BRDU kit; Phoenix Flow systems Inc.). Briefly, 2 million cells were seeded in 100 mm plates for 24 hours and treated with the indicated drugs for 24 hours to determine both the proportion of TUNEL-positive cells and the cell cycle phase from which apoptotic cells arose.

## Results

### Human ovarian carcinoma cells are sensitive to lovastatin-induced apoptosis

**Figure 1:**
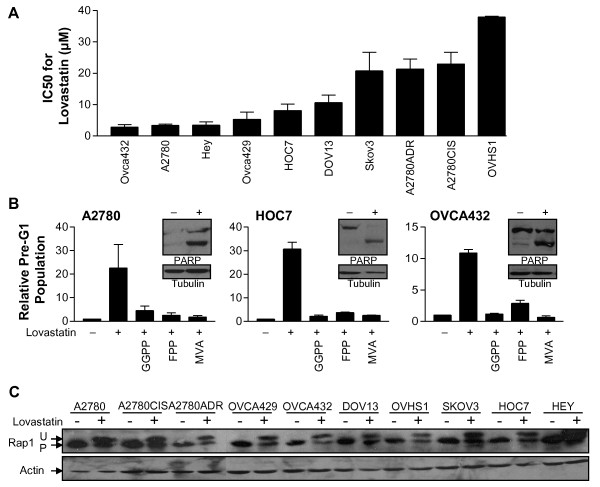
**Lovastatin triggers apoptosis in human ovarian cancer cell lines**. A series of experiments were conducted to define the cytotoxic effects of lovastatin on a panel of human ovarian cancer cell lines. A: Cells were exposed to a range of lovastatin concentrations for 48 hours to determine MTT_50 _values by MTT assay. MTT_50_s are presented as the mean of 2-4 independent experiments with error bars representing standard deviation. B: Cells were exposed to 20 μM lovastatin or vehicle control for 24 or 48 hours to assess the presence of PARP cleavage by western blotting. Tubulin was probed as a loading control. Experiments were conducted 2-3 times and representative blots are shown (see inset). For fixed PI, cells were seeded and treated as above to 20 μM lovastatin or vehicle control. Results are normalized to the ethanol controls (= 1%) and data are presented as the mean of 2-4 independent experiments with error bars representing standard deviation. C: Cells were seeded as above, exposed to 20 μM lovastatin or a vehicle control for 24 hours, lysed and immunoblotted to detect processed (P) and unprocessed (U) Rap1 and actin. Experiments were conducted 2-3 times and representative blots are shown.

To evaluate the sensitivity of human ovarian cancer cells to the anti-proliferative activity of statins, a panel of ten ovarian cancer cell lines was exposed to increasing concentrations of lovastatin for 48 hours. Lovastatin was the statin used in this study because it is readily available as a generic drug and is lipophilic, a feature of statins that have shown efficacy in recent breast cancer studies[25,26] MTT assays showed that lovastatin exposure triggered a substantial decrease in activity in all cell lines with MTT_50 _values that ranged from approximately 2 to 40 μM (Figure 1A).

To further define the antiproliferative effect of lovastatin, we next used two independent methods to evaluate whether lovastatin triggered apoptosis in the cell line panel. We first assessed the population of pre-G1 cells by fixed PI flow cytometry in response to 20 μM lovastatin for 48 hours. This fixed dose was used in all cell lines to be able to compare relative sensitivity. In all ten cell lines tested, an increase in the pre-G1 population was measured (Figure 1B, histograms, and data not shown). As an independent assay for apoptosis we assessed whether the cleavage of poly ADP-ribose polymerase (PARP) was detectable in cells treated with either lovastatin or vehicle control for 48 hours. Cleaved PARP was observed in all cell lines, except DOV13 (Figure 1B, inset western blots, and data not shown). Thus, ovarian cancer cells undergo apoptosis in response to lovastatin exposure.

Treating cells with 20 μM lovastatin for 48 hours elicited a robust apoptotic response from which a potent MVA-dependent rescue would be required. Cells were co-treated with lovastatin and either 100 μM MVA, 10 μM GGPP, or 10 μM FPP to determine if lovastatin-induced apoptosis in ovarian cancer cells is MVA-dependent. MVA reversed the effect of lovastatin and both GGPP and FPP were also able to partially rescue cells (Figure 1B, and data not shown). The mechanism of lovastatin-induced apoptosis in ovarian cancer cells is therefore dependent upon HMGCR inhibition.

To ensure that the MVA pathway block was due to lovastatin we evaluated the prenylation status of Rap1, a protein that is known to be exclusively geranylgeranylated (Figure 1C). Immunoblot analysis showed accumulation of the unprocessed form of Rap1 in all cell lines exposed to lovastatin for 24 hours, indicating that drug uptake was universally achieved.

### Lovastatin triggers apoptosis of human ovarian carcinoma cells in a time and dose dependent manner

**Figure 2:**
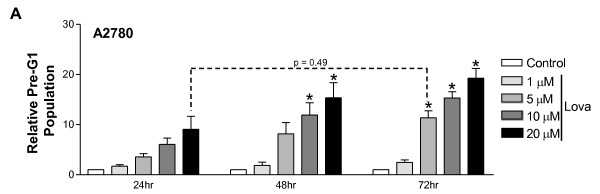
**Lovastatin-induced apoptosis of ovarian cancer cells occurs at therapeutically achievable levels**. A2780 cells were exposed to a range of lovastatin concentrations or vehicle control for 24, 48, or 72 hours to demonstrate the dose- and time- dependence of lovastatin-induced apoptosis as measured by fixed PI analysis. Results are normalized to each vehicle control (= 1%) and are presented as the mean of 3 independent experiments with error bars representing standard deviation. Dashed line highlights equivalence between doses of 20 μM for 24 hours and 5 μM for 72 hours. *, p < 0.05 compared to the untreated controls by a Student's t-Test.

Novel treatment options for women diagnosed with ovarian cancer are sorely needed. Our data suggests that statins have potential to be used as chemotherapeutics for these patients but it is important to determine whether they are effective at therapeutically achievable (low micromolar) concentrations. As a representative sensitive ovarian cancer cell line, A2780 cells were exposed to increasing concentrations of lovastatin (1, 5, 10, and 20 μM) for 24, 48, and 72 hours before being harvested for fixed PI analysis and the population of pre-G1 cells measured (Figure 2). Consistent with previous studies in AML, [11,27-30] the results clearly demonstrate that lovastatin-induced apoptosis of these cells is both dose- and time-dependent, and that the 20 μM, 24 hour lovastatin treatment commonly used in the laboratory is not significantly different (p = 0.49) from clinically achievable doses of lovastatin for 72 hours.

### Lovastatin-induced apoptosis of human ovarian carcinoma cells is p53-independent

**Figure 3:**
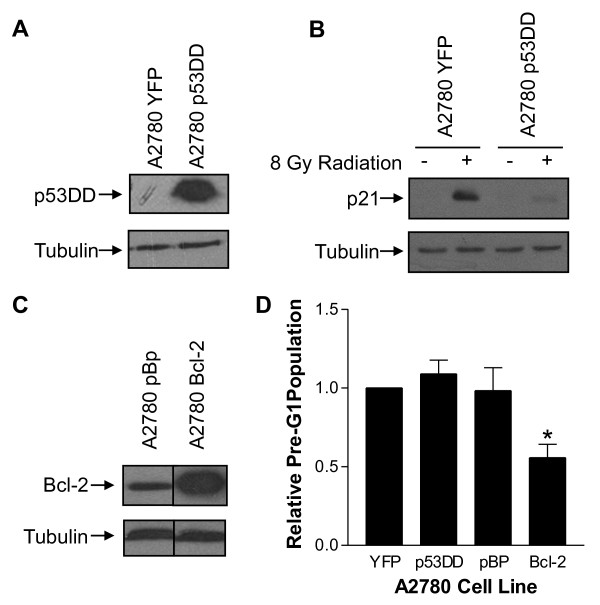
**Lovastatin-induced apoptosis in ovarian cancer cells is p53-independent**. A: A2780 YFP and A2780 p53DD cells were harvested, lysed, and immunoblotted with anti-p53 antibody to detect p53DD and with anti-tubulin as a loading control. B: A2780 YFP and p53DD cell were seeded and then exposed to 8 GY of radiation for 8 hours, harvested, lysed, and immunoblotted for the detection of p21 and tubulin. C: A2780 pBP and Bcl-2 cells were seeded as above, harvested, lysed, and immunoblotted with antibodies to Bcl-2 and tubulin. All experiments were conducted 2-3 times and representative blots are shown. D: A2780 YFP, p53DD, pBP, and Bcl-2 cells were seeded as above and exposed to 20 μM lovastatin or a control for 48 hours and analyzed by fixed PI to measure the pre-G1 population. Pre-G1 populations are expressed as ratios (lovastatin pre-G1/ethanol pre-G1) and normalized to the YFP vector control (= 1). Data are presented as the mean of 6 independent experiments with error bars representing standard deviation. *, p < 0.05 compared to the YFP control by a Student's t-Test.

To identify molecular features of cancers that predict sensitivity, we evaluated the role of the tumor suppressor p53 in lovastatin-induced apoptosis of ovarian cancer cells. Taking a molecular approach, a dominant negative p53 truncation, p53DD, was ectopically expressed in A2780 cells (Figure 3A). These cells are known to harbor endogenous wildtype p53 [31]. The activity of p53 in these cells was evaluated by exposing them to 8 GY of gamma-irradiation measuring p53-dependent induction of the cyclin-dependent kinase inhibitor, p21. As expected, p21 was induced after irradiation of parental and empty vector-expressing cells, but this induction was substantially decreased in A2780-p53DD cells (Figure 3B).

As a positive control for the inhibition of lovastatin-induced apoptosis, A2780 cells ectopically expressing Bcl-2 or the corresponding empty vector were also analyzed (Figure 3C). Fixed PI was used to measure the pre-G1 population of cells treated with 20 μM lovastatin for 24 hours (Figure 3D). Empty vector controls and cells expressing p53DD underwent a similar degree of apoptosis while ectopic expression of Bcl-2 significantly decreased the amount of lovastatin-induced apoptosis by 50%, demonstrating the p53-independence of lovastatin-induced apoptosis in human ovarian cancer cells.

### Lovastatin synergizes with doxorubicin in P-gp expressing cells

**Figure 4:**
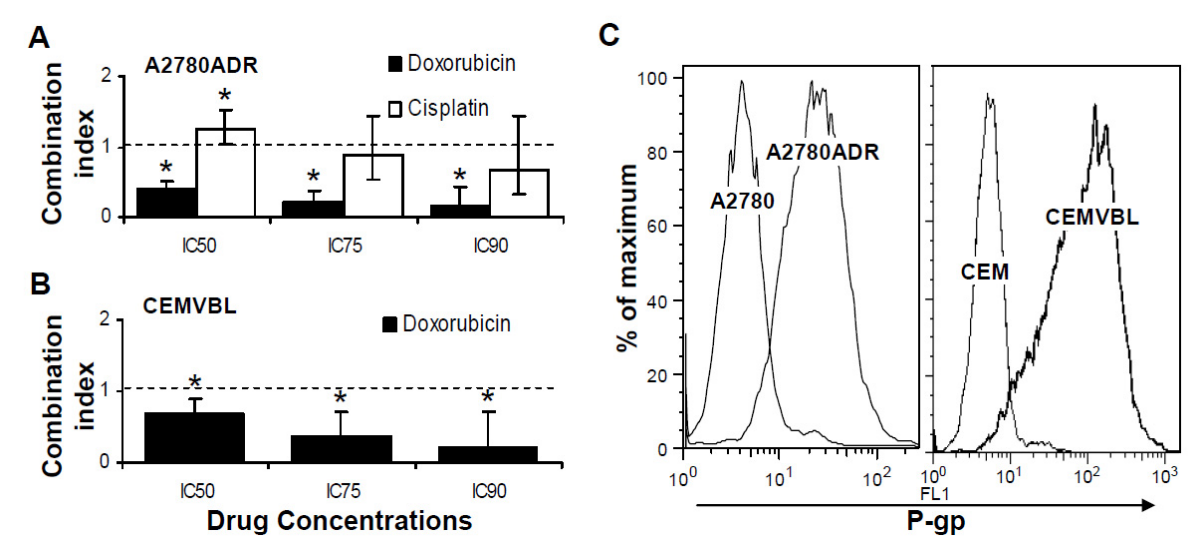
**Lovastatin synergizes with doxorubicin in P-gp expressing ovarian cancer cells**. A2780ADR (A) and CEMVBL (B) cells were plated for MTT assays and treated for 24 hours with concentration ranges of lovastatin and either doxorubicin or cisplatin. Combination index (CI) plots were generated using CalcuSyn software to determine whether the drugs synergize (CI < 1). Data is presented as the mean of 5-10 independently determined CI values with error bars representing standard deviation. *, p < 0.05 compared to additivity (CI = 1) by a Single Sample t-Test. C: Substantially higher P-gp expression was detected by flow cytometry in A2780ADR and CEMVBL cells compared to A2780 and CEM cells, respectively, using a fluorescence-tagged anti-P-gp antibody. The experiment was conducted 3 times; a representative histogram is shown.

As statins will likely be used in cancer treatment as part of drug cocktails, we next evaluated whether the addition of lovastatin to agents presently used in chemotherapy regimes of ovarian cancer would increase efficacy. Cisplatin and doxorubicin were selected as representative agents used in the treatment of primary and relapsed drug-resistant ovarian cancer. To model primary onset disease A2780 cells were treated in combination with cisplatin and lovastatin. To model relapsed disease A2780ADR cells, a multi-drug resistant cell line derived from parental A2780 cells, were treated with lovastatin in combination with either cisplatin or doxorubicin. Synergy experiments were adapted from the methods described by Chou and Talaly [21] to determine the antiproliferative effect of drug combinations with lovastatin. In A2780ADR cells, lovastatin and cisplatin were additive (C = 1) when combined at higher concentrations (Figure 4A, white bars). By contrast, lovastatin significantly synergized with doxorubicin in drug-resistant A2780ADR cells (CI < 1; Figure 4A, black bars).

A commonly proposed mechanism of multi-drug resistance (MDR) in recurrent ovarian cancer is elevated drug efflux, which is often due to increased activity of the ATP-binding cassette (ABC) transporter *ABCB1 *(previously named *MDR1*) gene that encodes P-glycoprotein (P-gp)[4] A2780ADR cells, previously developed by culturing parental A2780 cells in the presence of doxorubicin, have gained resistance to the drug by overexpressing P-gp, [22] which we confirmed by flow cytometry with a fluorescence-tagged antibody to P-gp (Figure 4C, left). Additionally, the MTT_50 _for doxorubicin determined by MTT assay in A2780ADR cells (4.9 μM at 48 hours; 3.1 to 7.7 95% CI) was approximately 100 times higher than in A2780 cells (0.04 μM at 48 hours; 0.03 to 0.06 95% CI). We hypothesized that P-gp mediated efflux of doxorubicin, a known substrate of P-gp, was being blocked by lovastatin via an unknown mechanism. To verify that synergy between lovastatin and doxorubicin was not simply an artifact of the A2780ADR cell system, we employed an alternative paired parental and MDR model derived from acute lymphoblastic leukemia, CEM and CEMVBL cells, respectively. We also confirmed that the CEMVBL cells both overexpress P-gp on their cell surface and have a significantly higher MTT_50 _for doxorubicin when compared to the parental CEM cells (Figure 4C, right, and data not shown). Interestingly, lovastatin synergized significantly with doxorubicin in CEMVBL cells (Figure 4B) using the same experimental design as above. We also determined that lovastatin did not synergize with cisplatin in either parental A2780 cells (Additional file 1: Supplemental Figure S1A) or the drug-resistant A2780CIS cells (Additional file 1: Supplemental Figure S1B), both of which had little to no P-gp expression compared to A2780ADR cells (Additional file 1: Supplemental Figure S1C). Furthermore, lovastatin and doxorubicin were borderline synergistic or additive in A2780 and A2780CIS (Additional file 1: Supplemental Figure S1) cells treated in a similar manner.

### Lovastatin increases doxorubicin retention in P-gp expressing cells

**Figure 5:**
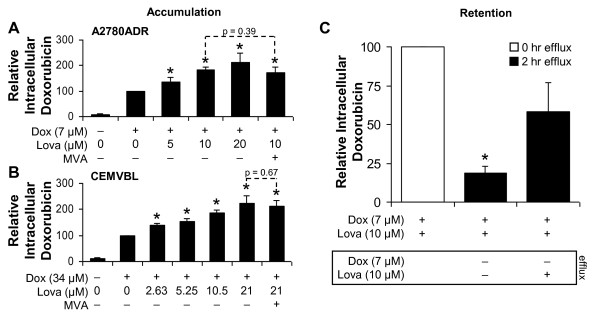
**Lovastatin increases doxorubicin retention in P-gp expressing ovarian cancer cells**. A2780ADR (A) and CEMVBL (B) cells were treated with lovastatin and doxorubicin as indicated for 3 hours, with or without MVA. Intracellular doxorubicin fluorescence was measured by flow cytometry and the results are normalized to cells treated with doxorubicin alone (= 100%). Data are presented as the mean of 3-4 independent experiments with error bars representing standard deviation. Dashed line highlights the lack of reversal by MVA. *, p < 0.05 compared to the 100% control by a Student's t-Test. C: A2780ADR cells were incubated with a combination of 7 μM doxorubicin and 10 μM lovastatin for 3 hours and were further incubated for two hours in doxorubicin-free media, with or without 10 μM lovastatin. Intracellular doxorubicin was determined by flow cytometry and the results are normalized to cells co-treated with doxorubicin and lovastatin without further incubation (= 100%). Data are presented as the mean of 3 independent experiments with error bars representing standard deviation. *, p < 0.05 compared to the 100% control by a Student's t-Test.

To elucidate the molecular mechanisms underlying this synergy we formulated a working model in which lovastatin blocks P-gp, thereby inhibiting its ability to drive the efflux of doxorubicin from MDR cells. As the fluorescence of doxorubicin can be directly measured by flow cytometry, we evaluated the amount of doxorubicin within A2780ADR and CEMVBL cells exposed to a sub-lethal dose of doxorubicin alone or in combination with increasing concentrations of lovastatin. Notably, A2780ADR (Figure 5A) and CEMVBL (Figure 5B) cells exposed to a combination of lovastatin and doxorubicin contained more intracellular doxorubicin than cells treated with doxorubicin alone. This process was dose-dependent, as increasing concentrations of lovastatin led to an increase in the accumulation of intracellular doxorubicin, but also observed at low physiologically relevant concentrations of both lovastatin (Figure 5A; 5B) and doxorubicin (data not shown).

Lovastatin also appears to prevent the active efflux of doxorubicin (Figure 5C). In this experiment, cells were treated with lovastatin and doxorubicin together to "load" the cells with doxorubicin. To determine differential degrees of doxorubicin retention, cells were further incubated for 2 hours in doxorubicin-free media with or without lovastatin. Remarkably, incubation with lovastatin resulted in more intracellular doxorubicin remaining after 2 hours. Partial loss of doxorubicin observed in cells that were incubated with lovastatin is likely due to passive diffusion or efflux mediated by alternative mechanisms because this same pattern was observed in parental A2780 cells, which do not overexpress P-gp, treated in the same manner (data not shown). These data suggest that lovastatin may inhibit P-gp from actively pumping doxorubicin out of the cell. Surprisingly, lovastatin-induced accumulation of doxorubicin was not reversed by co-incubation with MVA (Figure 5A; B), suggesting that a mechanism independent of HMGCR inhibition is at work. This data provides support for the combined use of lovastatin and chemotherapeutics that are substrates of P-gp to increase efficacy of tumor cell death.

### Combining lovastatin and doxorubicin potentiates DNA damage and apoptosis in P-gp expressing cells

**Figure 6:**
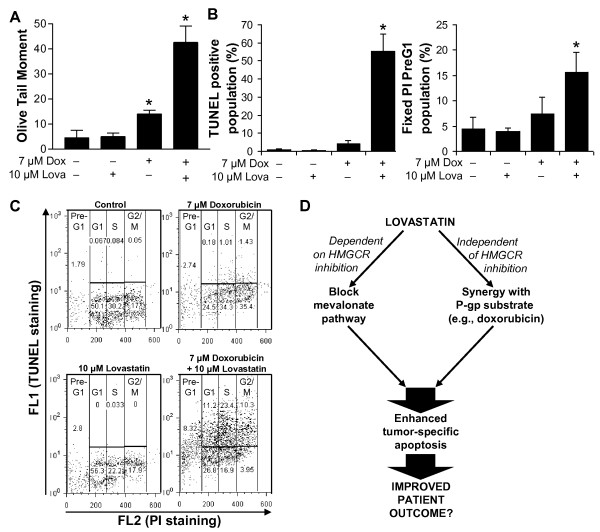
**Lovastatin potentiates DNA damage and apoptosis induced by doxorubicin**. A2780ADR cells were exposed to either a control, 10 μM lovastatin, 7 μM doxorubicin, or both 10 μM lovastatin and 7 μM doxorubicin together. A: Comet assays were performed and the olive tail moment determined for 75 cells of each condition. Data are presented as the mean of 4 independent experiments with error bars representing standard deviation. *, p < 0.05 compared to the untreated control by a Student's t-Test. TUNEL and PI dual-staining was carried out to determine the proportion of TUNEL-positive cells and if apoptotic cells originated predominantly in any particular phase of the cell cycle. B: Data are presented as the mean of 3 independent experiments measuring either TUNEL positivity (left) or PreG1 population (right) with error bars representing standard deviation. *, p < 0.05 compared to the untreated control by a Student's t-Test. C: Representative dot plots comparing the degree of TUNEL staining to DNA content. D: Schematic model illustrating two independent mechanisms of statin activity and the manner in which they can be combined to maximize clinical efficacy.

To further explore the mechanisms synergy between lovastatin and doxorubicin, we next measured DNA damage, commonly induced by doxorubicin, by comet assay. Drug concentrations used in this set of experiments were relatively sub-lethal, half-MTT_50 _values to minimize the effect of each drug on its own. Although these doses are higher than physiologically achievable levels, they remain experimentally tractable. While doxorubicin exposure alone resulted in a slight, significant increase in DNA damage compared to either control- or lovastatin-treated cells, combined treatment with both lovastatin and doxorubicin together resulted in a statistically significant 3-fold increase in DNA damage over doxorubicin alone (Figure 6A).

We next determined whether lovastatin could also potentiate doxorubicin-induced apoptosis. For these experiments we used dual-staining of TUNEL and fixed PI to measure the degree of apoptosis and determine if cells undergo apoptosis preferentially from any particular phase of the cell cycle. A2780ADR cells were treated as before and analyzed by flow cytometry. Similar to the comet assays, doxorubicin alone induced a small increase in apoptosis compared to either the control- or lovastatin-treated cells (Figure 6B). Cells treated with lovastatin alone, however, showed no evidence of either DNA damage or apoptosis. This is expected due to the low, sub-lethal dose used. Conversely, cells exposed to the combination of lovastatin and doxorubicin underwent a statistically significant 10-fold increase in apoptosis when compared to doxorubicin alone. While over-expression of Bcl-2 did not inhibit the combined activity of lovastatin and doxorubicin (Additional file 2: Supplemental Figure S2), apoptotic cells were detected from all phases of the cell cycle (Figure 6C). Therefore, doxorubicin and lovastatin combine synergistically to induce high levels of both DNA damage and apoptosis in human ovarian cancer cells.

## Discussion

Our work provides important evidence to support further pre-clinical and clinical evaluation of the statin family of drugs as anticancer agents against ovarian cancer. We show that a panel of ovarian cancer derived cell lines is sensitive to lovastatin-induced apoptosis, consistent with recent reports[14,15]. Mechanistically this apoptotic pathway is functionally blocked by exogenous MVA or the isoprenoid precursors GGPP and FPP. Moreover, we show that statin killing occurs irrespective of the mutational status of the tumor suppressor p53. Our results using a dominant negative p53 clearly indicate that lovastatin-induced apoptosis is substantially p53-independent and this is also supported by the observation that p53-null SKOV3 cells are able to undergo lovastatin-induced apoptosis. These observations are particularly important for ovarian cancer in which p53 mutation rates have been estimated between 23 and 79%[32]. We also show that lovastatin can synergize with doxorubicin and potentiate apoptosis. Synergy is achieved by lovastatin blocking drug efflux through a MVA-independent mechanism that enables the intracellular retention and genotoxic action of doxorubicin. To the best of our knowledge, these latter features of statin-induced apoptosis have not yet been reported for ovarian cancer. Exploiting the unique ability of statins to drive apoptosis by the mevalonate-dependent mechanism alone warrants further evaluation of these agents in the treatment of ovarian cancer (Figure 6D, left side). In addition, using statins, like lovastatin, to synergize with chemotherapeutics that are P-gp substrates (Figure 6D, right side) may be a feature of lovastatin action that further maximizes ovarian cancer cell death and improves patient survival.

It is interesting to note that while several reports have shown that P-gp expressing cells were more sensitive statin-induced apoptosis, [33-36] our results show the opposite trend (Figure 1A). Indeed, the MTT_50 _results for lovastatin in A2780ADR and A2780CIS cells are roughly 5-fold higher than in the parental A2780 cells. The reason for this difference is unknown, but it is possible that the drug resistant cells have exploited additional mechanisms of resistance, such as increasing the expression of anti-apoptotic proteins.

As agents approved for use in humans, the MVA-dependent antiproliferative activity of statins has prompted several Phase I clinical trials of statins on a wide variety of late-stage cancers, and although statins were well tolerated, only limited responses were evident. More recently statins have been evaluated on cohorts of patients harboring a tumor-type that has been shown to be sensitive to statin-induced apoptosis in tissue culture studies. In these focused, tumor-specific, hypothesis-driven trials, statins have demonstrated some efficacy as a single agent[29,30,37] but more wide-reaching effects were evident when statins were combined with chemotherapeutics [10,11,38,39]. Thus, our data identifying ovarian carcinoma as a statin-sensitive tumor type strongly supports the evaluation of statins in strategies to combat this disease.

A recent, retrospective epidemiological study showed that statin use in patients diagnosed with epithelial ovarian cancer is associated with improved survival[40]. Although only a relatively small number of patients met the criteria for the study, multivariable analysis identified statin use as an independent positive prognostic factor after controlling for age, stage, grade, and suboptimal cytoreduction, providing clinical support for the use statin-based combinations in cancer treatment. Similar recent analyses of breast cancer also provided additional insights. For example, it appears that lipophilic statin use after breast cancer diagnosis has been associated with decreased risk of recurrence[25,26]. Overall, these recent studies provide supporting rationale for the use of statins as anticancer agents and suggest that lipophilic statins (lovastatin, simvastatin, atorvastatin, fluvastatin, and pitavastatin) may be more effective, perhaps because they are better able to penetrate solid tumors compared to hydrophilic statins. From a pharmacological perspective, the lipophilic statins that demonstrate higher plasma concentrations with longer retention times in the circulation include atorvastatin and fluvastatin. This suggests these lipophilic agents may best target the tumor and show higher anti-cancer efficacy *in vivo*, consistent with a previous study comparing lipophilic and hydrophilic statins in ovarian cancer[15].

Recent evidence suggests that there may be a connection between drug resistance and regulation of the MVA pathway. In MDR AML cells, HMGCR mRNA levels were not significantly elevated upon statin exposure in cells that showed preferential sensitivity to lovastatin[36]. More recently, it was suggested that high levels of HMGCR mRNA correlates with sensitivity to statin-induced apoptosis[15]. It will be interesting in the future to determine how HMGCR expression impacts statin sensitivity and whether it can be exploited as a biomarker.

Mechanistically, it is clear that statins target HMG-CoA reductase and similarly trigger tumor cells to undergo apoptosis[7]. (Figure 6D, left side), however, several practical questions remain unresolved regarding statins as potential P-gp inhibitors (Figure 6D, right side). This new role of statins would be particularly important to consider in the management of ovarian cancer as survival and disease recurrence after taxane/carboplatin treatment has recently been associated with specific P-gp polymorphisms[41]. Several classes of specific P-gp inhibitors have been developed but have unfortunately shown general cytotoxicity in clinical trials[42]. This is thought to be due to targeting P-gp not only on tumor cells, but also on several normal vital organs that constitutively express P-gp. It would be easy to assume that statins blocking P-gp will similarly cause general cytotoxicity, however, it is not known whether statins and classic P-gp inhibitors are mechanistically or functionally similar. Lovastatin has been reported to inhibit P-gp in a limited number of biochemical studies with two very distinct caveats: none have used human cells overexpressing drug-selected human P-gp and the concentrations of drug used have been well beyond the physiologically achievable range [43-46]. Moreover, the results of these studies have been in conflict when using either the acid or lactone form of the statin[45,46]. Importantly, we conducted our work with physiologically attainable concentrations of both doxorubicin and lovastatin in human cell systems selected to overexpress human P-gp.

It is also worth noting that Bcl-2 was unable to inhibit cell death induced by the combination of lovastatin and doxorubicin (Additional file 2: Supplemental Figure S2). While the reasons for this result are unclear, it is possible that the cells have become drug-resistant through means other than the MDR machinery, such as upregulation of one or more anti-apoptotic proteins, and thereby rendered forced expression of Bcl-2 incapable of rescuing cells further. Further study will be required to better understand the interplay of all mechanisms of drug resistance.

Statins ultimately need to advance to clinical trials where their inhibition of drug efflux can be monitored on both tumor and normal cells. Interestingly, other groups have reported that lovastatin protects normal cells from doxorubicin-induced cytotoxicity [47-49] which, when combined with our data, suggests that statins may affect P-gp differently in normal cells compared to tumor cells. It is entirely possible that lovastatin functionally blocks P-gp in a manner that is distinct from classic P-gp inhibition. Evidence that statins can be successfully combined with various P-gp substrates is also established from their safe and effective combination in the polypharmacy of cardiac patients with hypercholesterolemia[50]. Taken together, our results suggest the ability of statins to trigger apoptosis of ovarian cancer cells may be exploited in the treatment of this disease, and that the potential P-gp inhibitory properties of certain statins, like lovastatin, warrant further investigation. It is also of interest to note that at MTT_50 _concentrations, but not higher, lovastatin had a slightly antagonistic relationship with cisplatin, a non-P-gp substrate (Figure 4A, Additional file 1: Supplemental Figure S1). This observation suggests that it could potentially be deleterious to combine lovastatin with cisplatin in the treatment of some patients. Furthermore, lovastatin and doxorubicin were also able to synergize in A2780 parental and A2780CIS cells. While this suggests that elements other than P-gp are involved in the interaction between these two drugs, the degree of synergy observed in A2780ADR cells is higher, indicating that inhibition of P-gp is likely an important mechanism of how lovastatin synergizes with doxorubicin. These results require further investigation to truly understand the manner by which lovastatin functionally interacts with other chemotherapeutics.

Determining which statin will maximally target different tumors, including ovarian, under different conditions will also be vital to advancing patient care. In the 14 completed and 20 or more ongoing clinical trials evaluating statins in the prevention or treatment of cancer, [10-12,30,37-39,51-57] the rationale for choosing a particular statin is not presented and appears random. Indeed, the ideal choice of statin as an anti-cancer agent remains unclear, however, evidence suggests lipophilic agents with pharmacologic properties that favor access to solid tumors is of high priority. Further work is required to better understand the activity of these statins as potential inhibitors of P-gp and to determine if this inhibition is specific to tumor cells *in vivo*.

## Conclusions

Overall, our results identify ovarian cancer cells as sensitive to statin-induced apoptosis and strongly suggest that statins can play a role in the treatment of ovarian carcinoma. As approved agents, statins can make immediate impact either as additions to traditional inductive therapy, as maintenance therapy to secure lasting remissions, or as salvage treatment for terminal, refractory disease. Our results may impact ongoing clinical trials using statins as anti-cancer agents and will be important to consider in the design of future clinical trials targeting various tumor types, including ovarian cancer.

## Competing interests

The authors declare that they have no competing interests.

## Authors' contributions

AM and JWC carried out experiments, participated in the design of the study, and contributed to drafting the manuscript. CAG carried out experiments. LZP conceived of the study, participated in its design and coordination, and helped to draft the manuscript. All authors read and approved the final manuscript.

## Pre-publication history

The pre-publication history for this paper can be accessed here:

http://www.biomedcentral.com/1471-2407/10/103/prepub

## Supplementary Material

Additional file 1 **Supplemental Figure S1**. Supplementary data that shows lovastatin did not synergize with cisplatin in either parental A2780 cells or drug-resistant A2780CIS cells and that lovastatin and doxorubicin were borderline synergistic or additive in A2780 and A2780CIS cells. Click here for file

Additional file 2 **Supplemental Figure S2**. Supplementary data that shows Bcl-2 was unable to inhibit cell death induced by the combination of lovastatin and doxorubicin. Click here for file
