# Supplementary material for: Lovastatin induces apoptosis of ovarian cancer cells and synergizes with doxorubicin: potential therapeutic relevance
Source: BMC Cancer. 2010 Mar 18;10:103. doi: 10.1186/1471-2407-10-103 (PMC2847546; doi:10.1186/1471-2407-10-103)
Supplement: Additional file 1 — Supplemental Figure S1. Supplementary data that shows lovastatin did not synergize with cisplatin in either parental A2780 cells or drug-resistant A2780CIS cells and that lovastatin and doxorubicin were borderline synergistic or additive in A2780 and A2780CIS cells. [file 1471-2407-10-103-S1.PPT]

## Slide 1
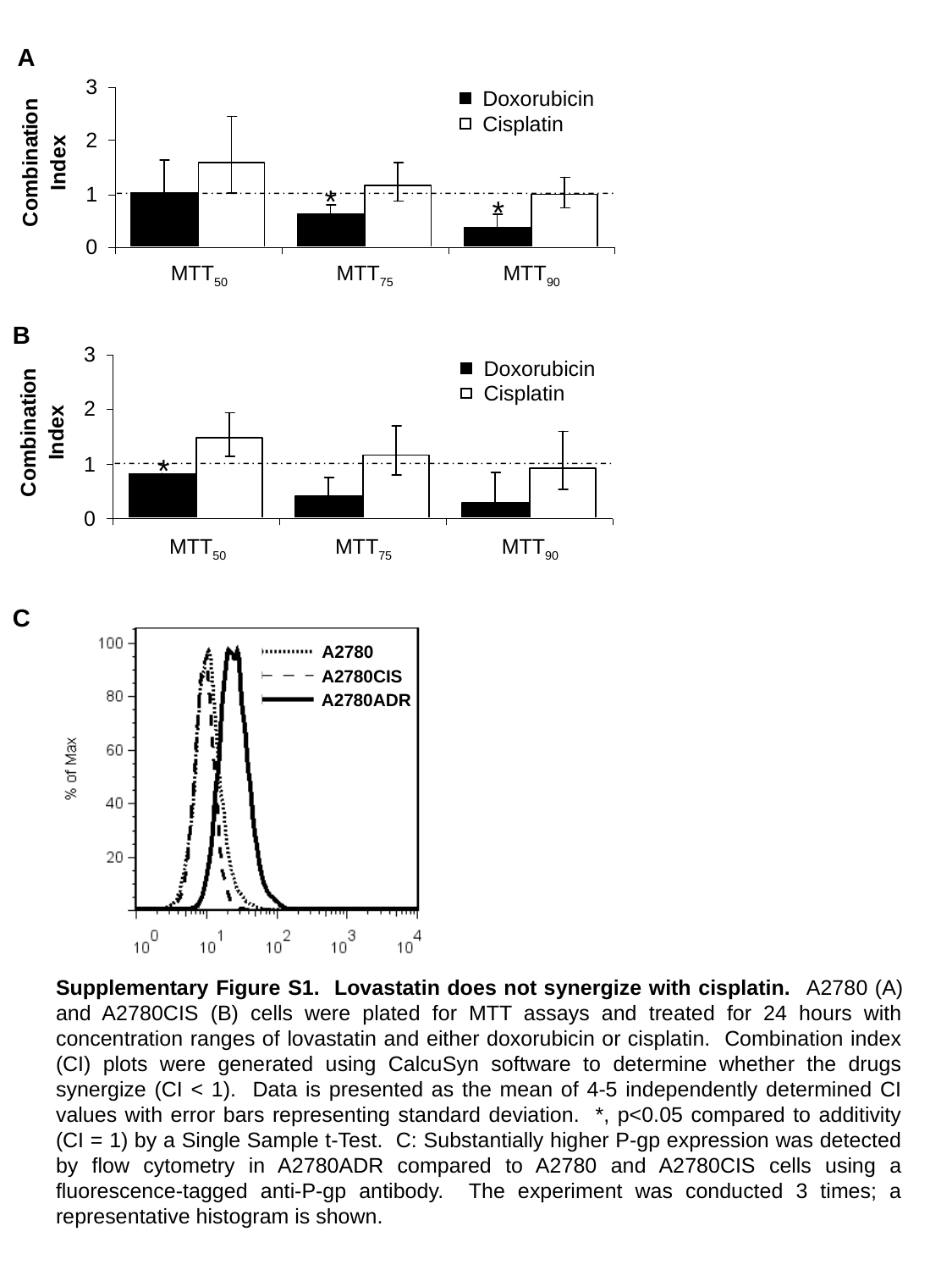

A
Doxorubicin
Cisplatin
*
*
MTT50
MTT75
MTT90
B
Doxorubicin
Cisplatin
*
MTT50
MTT75
MTT90
C
A2780
A2780CIS
A2780ADR
Supplementary Figure S1. Lovastatin does not synergize with cisplatin. A2780 (A) and A2780CIS (B) cells were plated for MTT assays and treated for 24 hours with concentration ranges of lovastatin and either doxorubicin or cisplatin. Combination index (CI) plots were generated using CalcuSyn software to determine whether the drugs synergize (CI < 1). Data is presented as the mean of 4-5 independently determined CI values with error bars representing standard deviation. *, p<0.05 compared to additivity (CI = 1) by a Single Sample t-Test. C: Substantially higher P-gp expression was detected by flow cytometry in A2780ADR compared to A2780 and A2780CIS cells using a fluorescence-tagged anti-P-gp antibody. The experiment was conducted 3 times; a representative histogram is shown.
