# Supplementary material for: Lovastatin induces apoptosis of ovarian cancer cells and synergizes with doxorubicin: potential therapeutic relevance
Source: BMC Cancer. 2010 Mar 18;10:103. doi: 10.1186/1471-2407-10-103 (PMC2847546; doi:10.1186/1471-2407-10-103)
Supplement: Additional file 2 — Supplemental Figure S2. Supplementary data that shows Bcl-2 was unable to inhibit cell death induced by the combination of lovastatin and doxorubicin. [file 1471-2407-10-103-S2.PPT]

## Slide 1
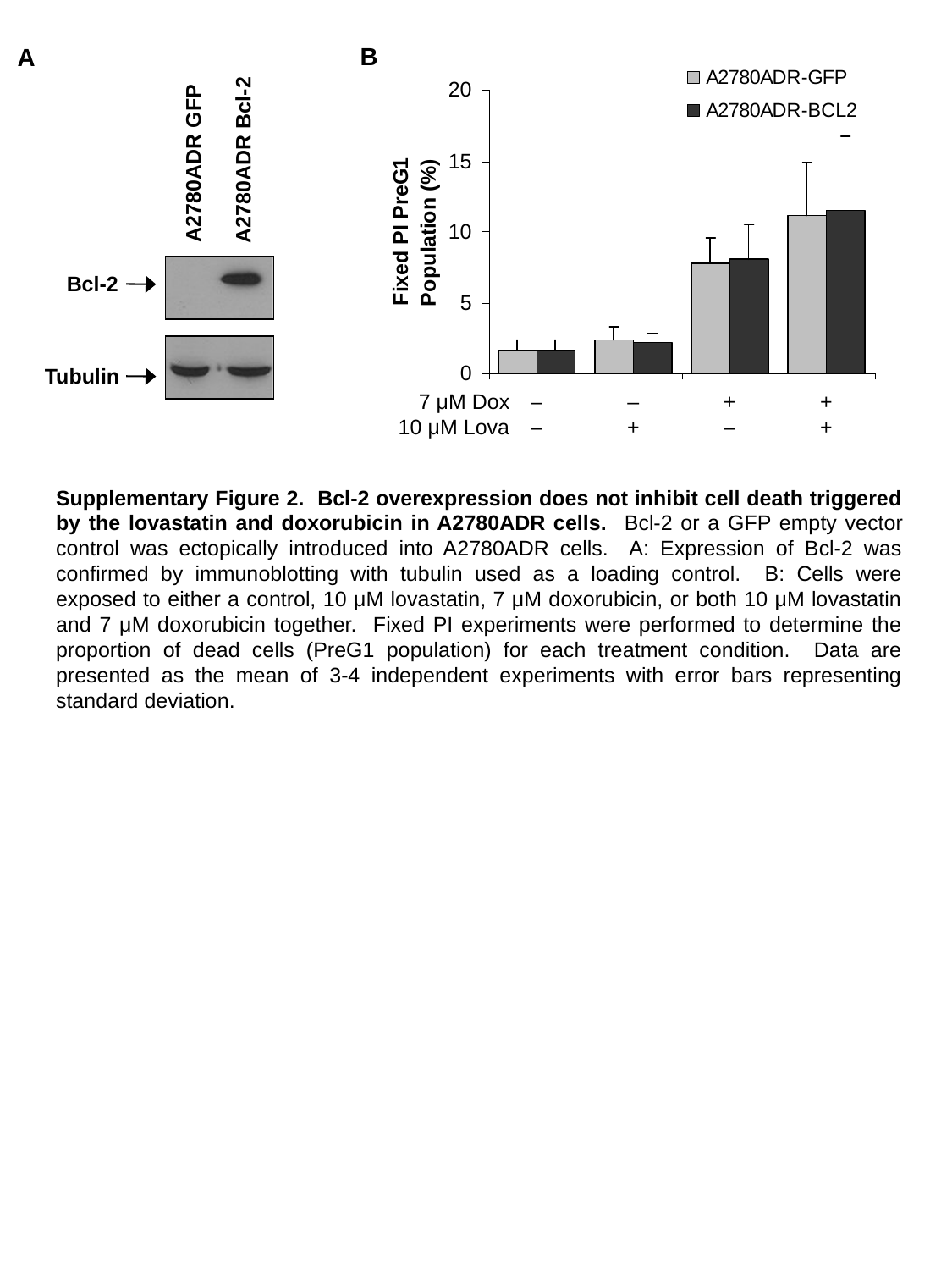

B
A
7 μM Dox
10 μM Lova
–
–
–
+
+
–
+
+
A2780ADR Bcl-2
A2780ADR GFP
Bcl-2
Tubulin
Supplementary Figure 2. Bcl-2 overexpression does not inhibit cell death triggered by the lovastatin and doxorubicin in A2780ADR cells. Bcl-2 or a GFP empty vector control was ectopically introduced into A2780ADR cells. A: Expression of Bcl-2 was confirmed by immunoblotting with tubulin used as a loading control. B: Cells were exposed to either a control, 10 μM lovastatin, 7 μM doxorubicin, or both 10 μM lovastatin and 7 μM doxorubicin together. Fixed PI experiments were performed to determine the proportion of dead cells (PreG1 population) for each treatment condition. Data are presented as the mean of 3-4 independent experiments with error bars representing standard deviation.
